# Emergence of online communities: Empirical evidence and theory

**DOI:** 10.1371/journal.pone.0205167

**Published:** 2018-11-14

**Authors:** Yaniv Dover, Guy Kelman

**Affiliations:** Hebrew University, Jerusalem, Israel; Tampereen Yliopisto, FINLAND

## Abstract

Online communities, which have become an integral part of the day-to-day life of people and organizations, exhibit much diversity in both size and activity level; some communities grow to a massive scale and thrive, whereas others remain small, and even wither. In spite of the important role of these proliferating communities, there is limited empirical evidence that identifies the dominant factors underlying their dynamics. Using data collected from seven large online platforms, we observe a relationship between online community size and its activity which generally repeats itself across platforms: First, in most platforms, three distinct activity regimes exist—one of low-activity and two of high-activity. Further, we find a sharp activity phase transition at a critical community size that marks the shift between the first and the second regime in six out of the seven online platforms. Essentially, we argue that it is around this critical size that sustainable interactive communities emerge. The third activity regime occurs above a higher characteristic size in which community activity reaches and remains at a constant and higher level. We find that there is variance in the steepness of the slope of the second regime, that leads to the third regime of saturation, but that the third regime is exhibited in six of the seven online platforms. We propose that the sharp activity phase transition and the regime structure stem from the branching property of online interactions.

## Introduction

Peer-to-peer group interactions are prevalent in online platforms. People regularly participate in online groups and communities, interact with other members, and are affected by their peers [1–4]. Still, there is little empirical evidence that pins down the factors that determine whether a community will keep thriving with activity or fail to attract, or retain, active members. The extant literature discusses several factors that are important in maintaining meaningful social group action [5–11]. First and foremost are the number of committed group members at a given time. This is a prominent indicator of an active community, even if the commitment levels are heterogeneous [5]. The second factor is the minimal level of interdependence required between group members to induce any interaction within the community [12, 13]. Third, the marginal returns on contribution should be non-decreasing [5]. Other factors such as group context and social network structure [14] have also been surveyed in the theoretical literature, but some studies [8] suggest that these effects are “second order.” Here, we wish to gain empirical insight into online communities, stability by investigating the relationship between activity and size. It is not immediately clear from the literature what the expected exact activity–size relationship should be, although some studies suggest that this relationship should strongly depend on the underlying production function, context, competition [15, 16], and heterogeneity [5, 11].

Because our exploration and data is extracted from a variety of contexts in the online world, it is important to clarify what we mean by “online community.” Note, also, that the term “community” is usually used in the context of the network analysis and community detection literature (e.g., [17]), which is not the meaning we ascribe to it here. A robust definition of the term “online community” has been the subject of heated debate and controversy, since the emergence of computer-mediated communications (see, e.g., [18] for an overview). In some instances, online communities are defined by ad hoc community-life parameters (e.g., shared interests, self-reported group affiliation), but a prevalent and more general approach in the literature is to define groups of people as communities if member-to-member interactions exist within it, i.e., that there is some sort of social network of interactions between members (e.g., [19, 20]). A more detailed definition is suggested in [21] and [22]: “An online community is a group of people, who come together for a purpose, online, and who are governed by norms and policies and supported by software.” Notably, this definition includes weaker forms of interaction, such as interaction around specific tasks or content, which are also a subject of investigation and interest in the literature. Here, we will use the term “online community” to denote a group of people who interact digitally around a common theme, or purpose, within a relatively confined and defined context. The definitions we cite consider communities to be separate and distinct groups of people. But, our data is secondary and extracted from the online world and, therefore, we have to make a simplifying assumption. Throughout the paper, we assume that when a group of people congregates around a common theme (e.g., within a topical discussion forum or around a piece of online content), they form for this purpose an ad-hoc community that is separate from other communities on the platform. We ignore, in that sense, the fact that within and across platforms, the membership of communities may overlap. We hope that future research will tackle this distinction and explore the consequences of making this assumption.

For example, a group of users in a specific discussion forum will be considered an online community because they are a group of people who interact within the interface confines of the forum and, potentially, around its proclaimed theme. In that same spirit, authors of a Wikipedia page timeline interact with one another around the common goal of creating and maintaining the page. Commenters on a YouTube video page also, presumably, interact around the theme of the video. In some of these contexts, the social interactions may be considered weak, in others, more intensive. Generally, the communities we explored all fall under the above definition of “online communities.”

To investigate the relationship between activity and size across a variety of types of online communities, we collected and analyzed several rich datasets that contain hundreds of thousands of online communities, spanning a time frame of more than a decade. (cf. the Materials and methods section that gives detail of the data collection and processing across platforms). In what follows, we detail the patterns observed in the relationship between community activity and community size.

## Results

### Activity–size phase transition and regime structure

**Fig 1:**
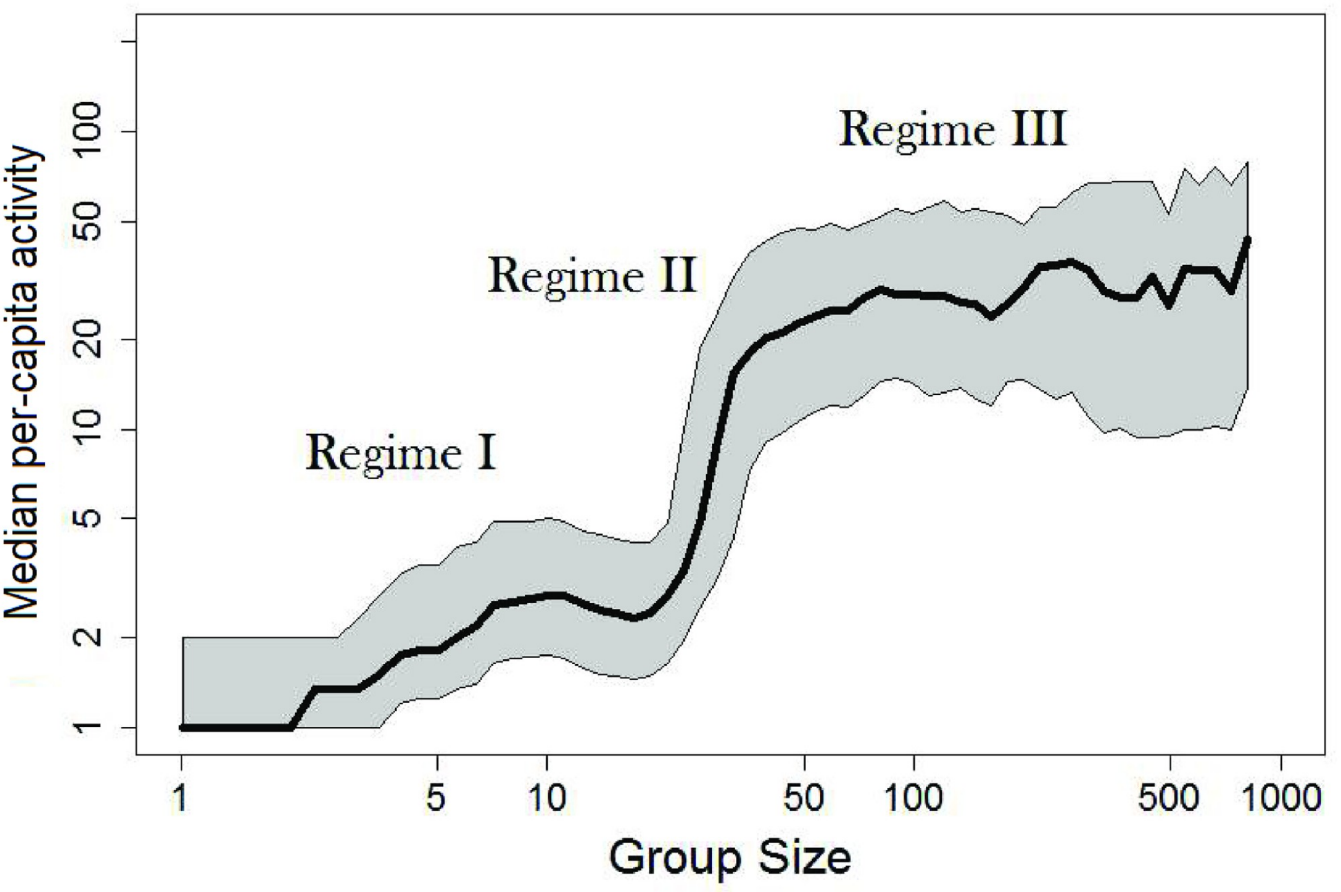
Mean activity across a range of community sizes in the TAP (“Tapuz Communes”) data. The thick solid line visualizes the median activity across community-size bins. The shaded areas mark the regions between the 25th to 75th percentiles.

Fig 1 shows the median per-capita activity of online communities as a function of their size for a large website that hosts thematic discussion-based online communities (TAP dataset, see Methods for details). On this platform, users can choose to either initiate or terminate their own communities, which allows us to observe their “organic” life cycle across time. We use per-capita activity, i.e., mean user activity, to control for linear size effects. Fig 1 shows a striking activity–size phase transition and three distinct size regimes of activity that can be summarized as follows:

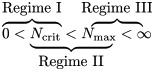


*Regime I* spans small communities of up to about 20 members. In this regime, activity is sporadic, and, across the lifetime of a community, the mean number of posts per user is low, at around two posts per user. The slope of the dependence of mean activity on community size is 0.086 (with a std. error of 0.018). Analysis shows that, in this regime, a community requires more than 12 new users just to “encourage” community members to increase their posting rate by one additional message. Thus, the effect of community size on participation is very small. A sharp transition to *Regime II* occurs in communities of about 20 members, and ranges up to group sizes of about 50. This type of sharp transition around a critical mass, which we denote here by *N*_crit_, is theorized in the literature [23], but empirical evidence for actual relevant examples are scarce. Below, in the Model section, we explain how this sharp transition likely results from the branching property of discussion trees. In short, activity within an online community is essentially a collection of messages embedded within interaction (discussion) trees.

These trees of interaction can either grow multiplicatively, if there is a minimal number of members willing to interact, or they can remain shallow and limited if the number of members is small and the members are non-responsive. Consequently, the trees’ branching property creates a situation in which small variations around a critical community size, will result in a dramatic difference between a regime with rare interactions and one in which interactions are abundant such that discussion trees can grow exponentially. The activity–size slope within *Regime II* is 0.91 (SE 0.085), meaning that it only takes one additional user to the community to be associated with an increase of one additional message to the posting rate of the typical user. This effect is an order of magnitude greater than in *Regime I*. In other words, a meaningful effect of community size on participation emerges in *Regime II*. The transition into a third regime, *Regime III*, takes place in communities that roughly number 50 members or more. Here, like in *Regime I*, the slope is very small (0.022, SE 0.002). It seems, therefore, that there is a cap on community effects above a certain size, which we denote by *N*_max_. In the Model section, we estimate *N*_max_ and *N*_crit_ among other parameters.

**Fig 2:**
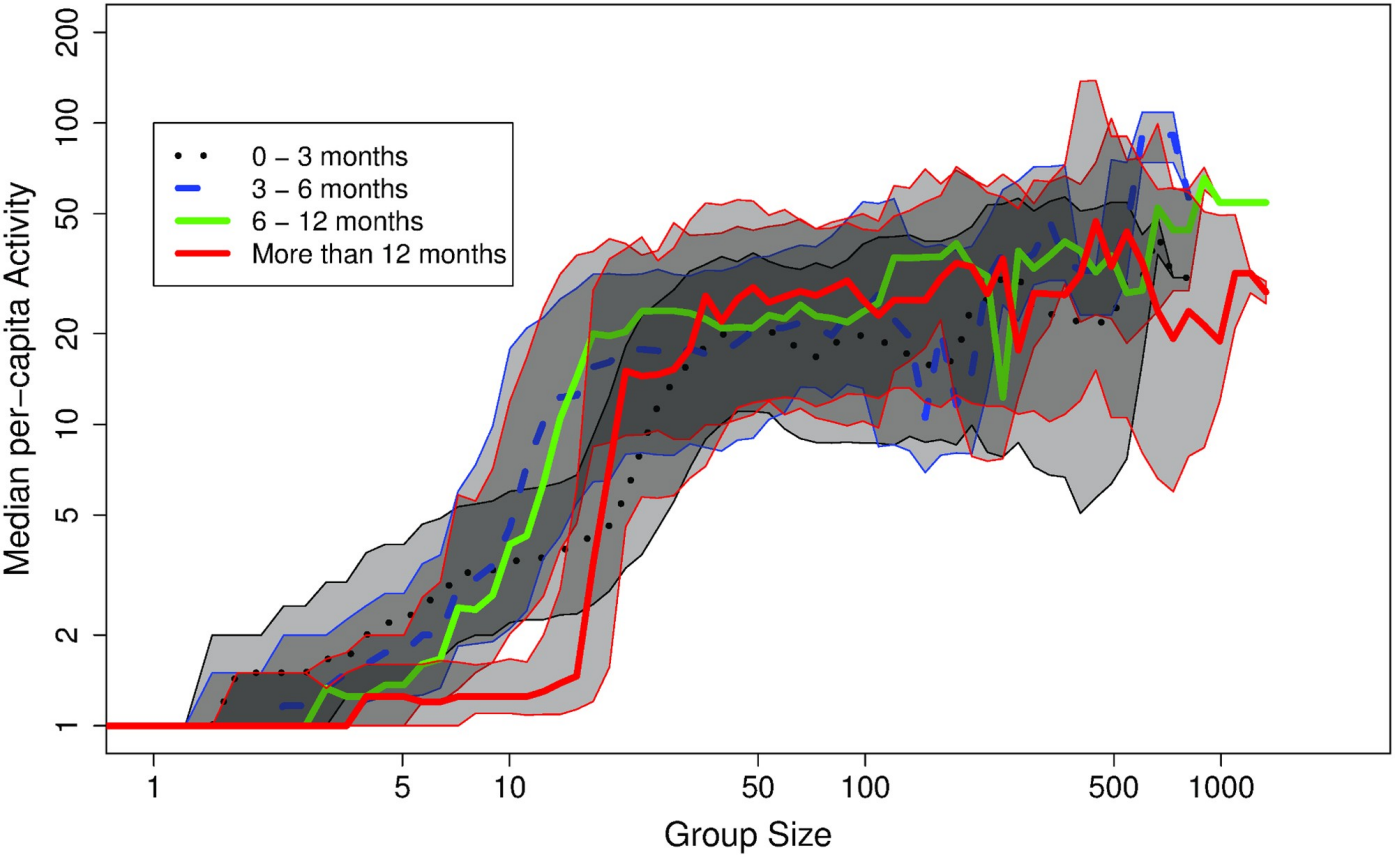
Mean activity across community sizes in the TAP data, for varying life stages in communities’ lifetimes. (a) younger than 3 months (black dotted curve), (b) between three and six months (blue dashed curve), (c) between six months and a year (green solid line) and (d) more than a year (red solid line).

Notably, Fig 1 summarizes the dynamics of communities over their entire lifetime. A lifetime in these data can span a few weeks or a few years, depending on activity levels and the time point at which activity ceases. In order to rule out a scenario where the three-regime structure is an artifact of some complex long-term dynamics, Fig 2 shows the same empirical dependency, but for varying community lifetime stages. Communities were divided into four groups of lifetime brackets so that the sample sizes in each group are large enough to exhibit a full size–activity curve. Very young communities, of up to three months of activity, are denoted by the black dotted curve, mid-life communities are denoted by the blue dashed curve (three to six months) or solid green curve (six to twelve months), and the oldest communities which have existed for more than a year, are denoted by a solid red line. Notably, communities that are three months or older exhibit a distinct three-regime pattern. While mid-life communities show a high slope in *Regime II*, similar to that exhibited in Fig 1, the oldest communities show an even-steeper slope. On the other hand, for very young communities the sloping transition from *Regime I* to *Regime II* and *Regime III* is smoother. Our interpretation of this is that even though the regime structure exists throughout an online community’s life cycles, the older the community is, the stronger the phase activity–size phase transition. While we can only speculate, we assume that a possible explanation is that, for various reasons discussion trees occur more efficiently in older communities; therefore, the phase transition between *Regimes I* and *II* is more pronounced. While this development explanation may be appealing in the case of the TAP dataset, we do not see consistent patterns in other data (see S1 File for more detail).

Another concern is that *Regime II* may be a spurious result of, in fact, two regimes, I and III, between which there is a discontinuous jump, but with heterogeneous critical points. First, this scenario is not consistent with the results shown below, in which all three regimes are exhibited even for narrow ranges of critical points. Second, when inspecting the distribution of jumps in the activity of communities, it turns out that only 8.6% of them exhibit a discontinuous jump in their mean activity of > 10 posts per member, and only 2.9% of communities show a jump of activity that is higher than 20 posts per member. In contrast, the gap between *Regime I* and *Regime III* (e.g., in Fig 1) is larger than 20. Therefore, it is not probable that the observed *Regime II* is spurious, i.e., a result of heterogeneity of critical points.

### Activity–size phase transition is also exhibited in discussion trees structure

**Fig 3:**
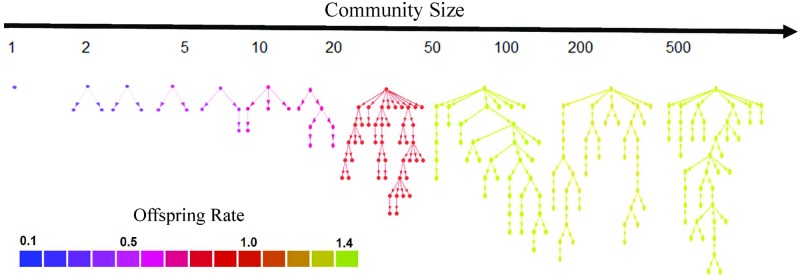
A schematic of selected discussion trees ordered by community size. The nodes mark response posts and the root is the initial “seed” post. Links between nodes are the association of post and reply. The illustrated trees are color-coded according to the mean rate of replies to messages (mean offspring rate) within each community size tier.

Online peer-to-peer interactions can be thought of as trees of messages and replies (see for example, [24]). In these trees, messages are nodes and are connected by links that represent which message was addressed as a reply to which other previous message. This discussion tree begins with an initial seed message posted by a user. Other users can then post a reply to the seed message, i.e., link their messages to the initial one, creating a two-level tree. This tree can branch out further with replies to the replies at deeper levels, and so on. As we outline in the Model section, the rate of growth of a discussion tree depends solely on the distribution of the number of offspring, or replies to each message. If the mean number of replies per message is higher than one, the tree grows multiplicatively. If the mean number of replies is lower than one, the growth of the tree effectively decays geometrically. Therefore, we expect a sharp phase transition of discussion tree sizes, or number of messages, to occur as the mean response rate increases linearly within a community. We argue that the phase transition observed in Fig 1, across communities, stems from this branching property of peer-to-peer discussion trees. As a community grows in size, response rates also grow, and above the critical point of one reply per post, a sharp phase transition of multiplicative tree growth occurs. Evidence that this is indeed the actual scenario can be seen in Fig 3. This figure sketches typical discussion trees sampled from the data and arranged by community size. Each displayed tree is representative of the median depth at the given community size in the TAP dataset. The figure shows that the activity phase transition between *Regime I* and *Regime II* is strongly correlated with a sharp increase of tree depth. The mean response rate (*q* ⋅ *N*) at a given community size, is also displayed in Fig 3 as the color of the illustrated tree. Consistent with our theory (see Model section), the figure does show that the transition occurs around the offspring rate of unity.

### The relationship between responsiveness and critical community size

An outcome of the suggested theory is that a community’s critical size strongly depends on community responsiveness. A crude but useful approximation for this relationship can be seen under simplifying assumptions: $N_{\text{crit}}≃\frac{1}{q}$, where *q* is the community mean level of responsiveness (see the Model section). This approximation emerges from the requirement that the mean response rate at the critical point will be unity, i.e., *q* · *N*_crit_ ≃ 1. Our data are at the individual level, so it is possible to estimate the responsiveness *q* for sets of communities and to investigate the dependence of *N*_crit_ on *q*. First, the analysis shows that the most common responsiveness in the TAP data is *q* ≅ 0.04. This translates to an estimated *N*_crit_ = 25, which is roughly the actual transition point observed in Fig 1.

**Fig 4:**
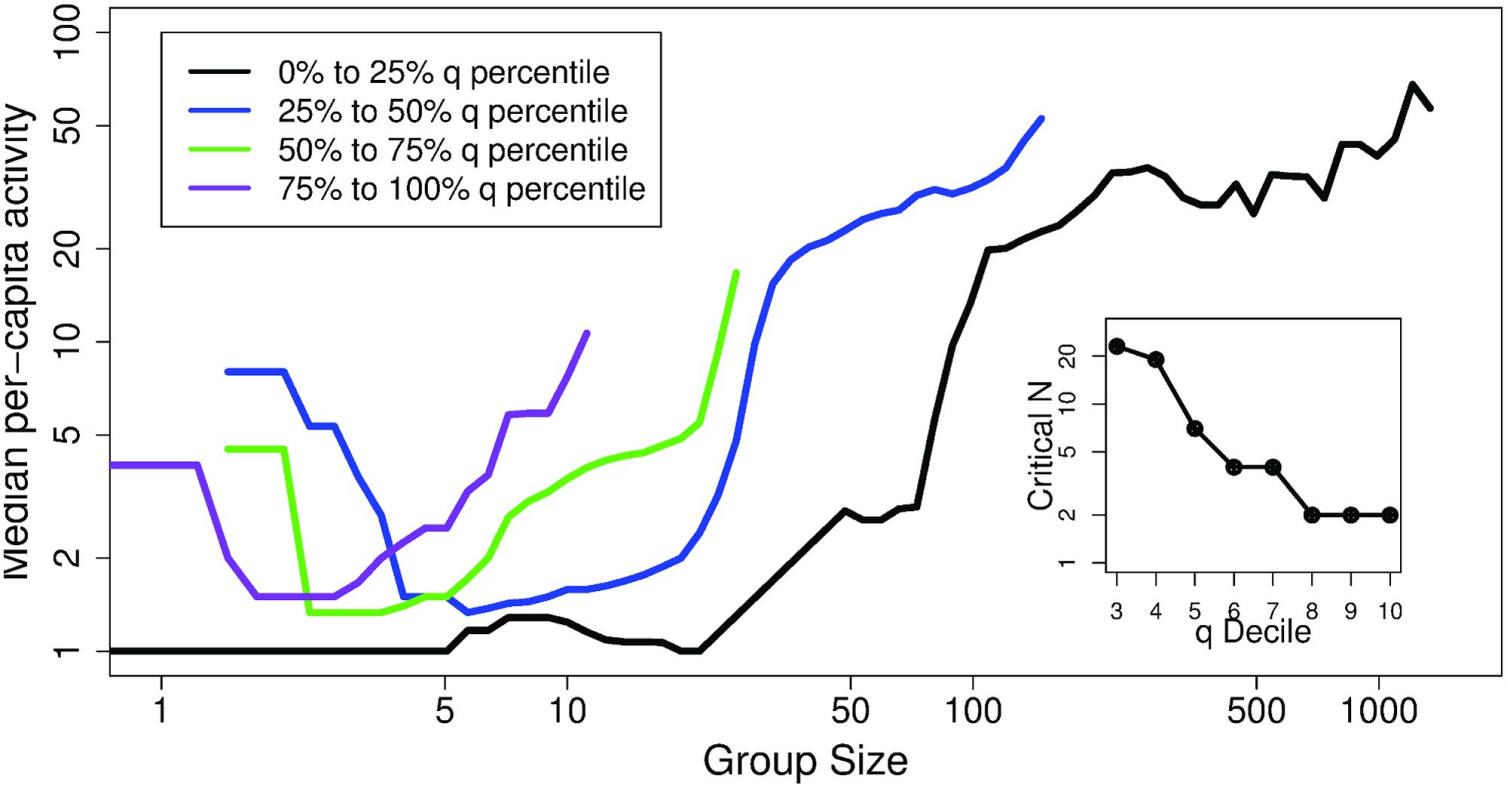
Activity median as a function of community size for communities grouped in four quartiles of response rate q (see legend for details). The inset shows, for a ten-fold partition of *q* values (deciles) the *N*_crit_ as a function of within-decile *q*.

Another test of the branching message tree theory is whether communities with higher responsiveness rates will correspond to lower critical community sizes. Unfortunately, an inherent property of these data is that the subset of communities with homogeneous *q* is small. This limits our ability to accurately validate the relationship $N_{\text{crit}}≃\frac{1}{q}$, but it is still possible to test whether *N*_crit_ decreases with increasing *q*, as we expect. To do this, we divide the data into four equal-count bins representing the quartiles of *q* values, from low to high. Fig 4 shows the mean activity versus size curves for each of the four quartiles. The figure indicates that a sharp transition does exist for each subgroup and that the *Regime I*–*Regime II* transition shifts continuously leftward (*N*_crit_ decreases) as the within-bin *q* increases. For comparison, the expected *N*_crit_ values that were calculated using the mean *q* within each bin are: 1343.7, 37.7, 15.6, and 5.2, respectively for the first, second, third, and fourth *q* bins. These estimations seem to be roughly consistent with the order of magnitude of the transition points in Fig 4. This is encouraging given the within-bin sample size limitation, heterogeneity within bins, and the crudeness of the approximation. Calculations at higher resolution, i.e., with 10 bins of *q* (deciles) are shown in the inset of Fig 4 and support the expected reciprocal relationship between *N*_crit_ and *q*.

### Activity–size patterns across platforms: Do they share common features?

**Fig 5:**
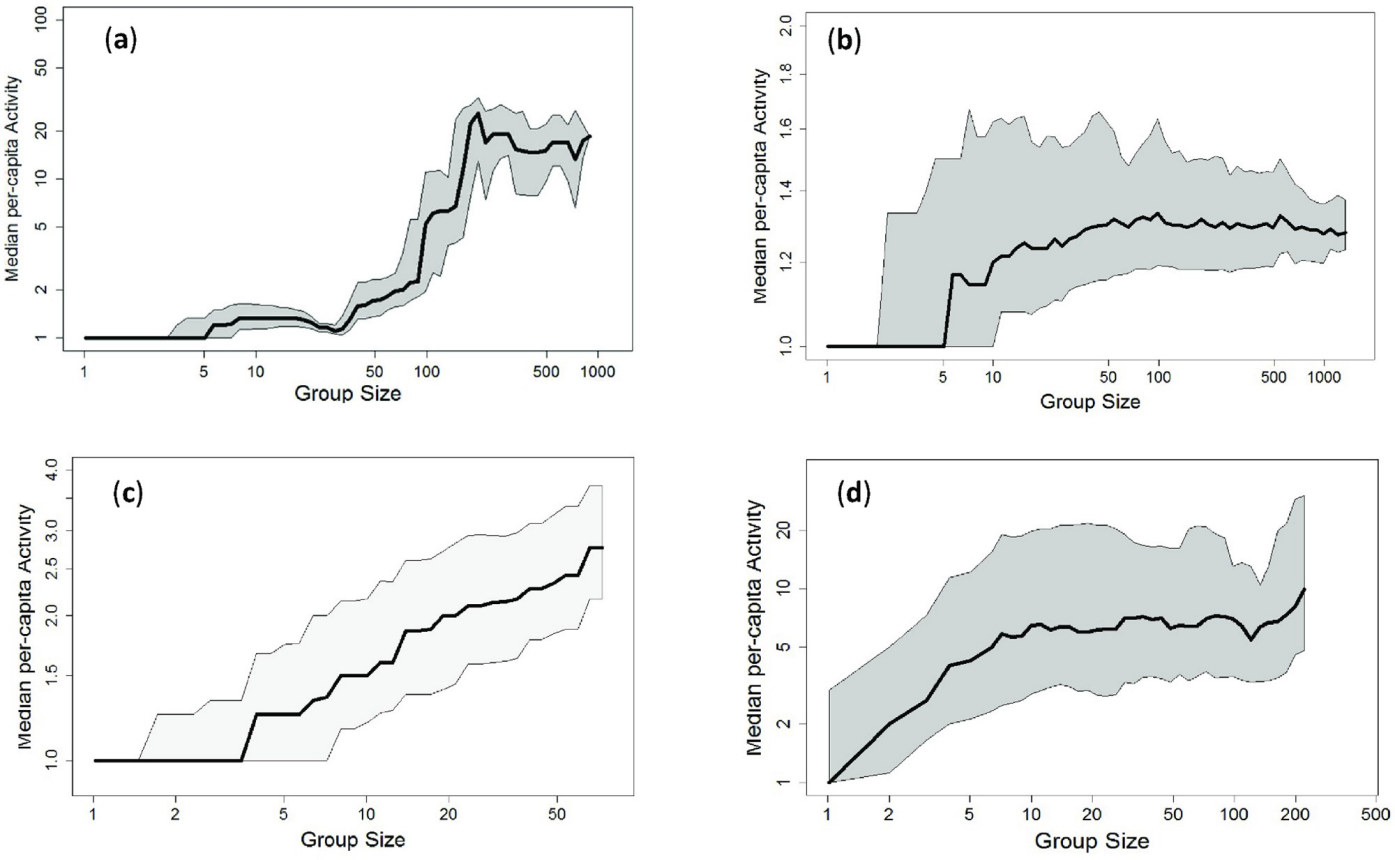
Activity median as a function of community size for other platforms. (a) Boards.ie (BRDS), (b) YouTube (YOUT), (c) Wikipedia (WIKI) and (d) Goodreads (GOODR). For visualizations of other platforms see Figs A and B in S1 File.

To test whether the three-regime pattern is unique to the TAP platform or is a more general phenomenon, we collected data from six additional online platforms. For consistency, we chose platforms that enable users to post messages and replies within distinct predefined communities (see the Methods section). The activity–size profiles for the additional platforms are laid out in Fig 5 and in S1 File.

Panel Fig 5(a) illustrates the median activity as a function of community size for BRDS, the public dataset of Boards.ie. Much like the TAP set, BRDS has a distinct three-regime pattern. In this case, however, *N*_crit_ is roughly 100, suggesting that the average community’s ambient responsiveness is smaller on this platform, around $q≃\frac{1}{100}$. Panel Fig 5(b) displays comment discussions from 8,446 random YouTube video pages. Note that, in this case, there are some differences from the TAP and BRDS datasets. While we still observe all three regimes and a sharp phase transition between *Regimes I* and *II*, the maximum activity in the third regime (i.e., above *N*_max_) is not as high as it is for TAP and BRDS. We speculate that this is likely because YouTube is not specifically used for lengthy and continuous peer-to-peer discussions. In fact, the platform only allows one level of replies to posts. While this does not prevent users from continuing to reply to one another, it potentially inhibits tree growth and activity levels. The graph and patterns for HI5 and RED (Figs A and B in S1 File) are similar to the YOUT case, and are shown in S1 File.

Next, Panel Fig 5(c) shows user discussions in a random collection of 21,000 Wikipedia talk pages. Here, a main difference of this case from the others is that we do not observe a third regime. A possible explanation is that, in this platform, community sizes are typically small [25], and substitution of members often supersedes growth. For our data, size does not exceed 80 users per community, and the median is *N*_authors_ < 10, so it may be the case that communities large enough to exhibit activity saturation, do not exist. The transition between *Regimes I* and *II* occurs in communities consisting of about 3–4 authors, suggesting high responsiveness. Lastly, Panel Fig 5(d) shows the activity profile of 8,040 Goodreads discussion communities randomly collected and analyzed. Although *Regimes II* and *III* are distinguishable in this panel, *Regime I* is missing. This is a possibility that is also captured in our tree discussion model. It occurs in the case of high levels of responsiveness. If responsiveness is high enough, the critical point will be so low that *Regime I* will be too small to be observed. In general, it is important to note that our branching model, outlined in the next section, inherently accommodates the scenario in which *Regime I* or *Regime III* are not observed. Low values for critical size are expected for tightly-knit or highly interactive communities in which the responsiveness is high. The occurrence of *Regime III* depends on whether communities reach sizes of the order of *N*_max_ and grow beyond them. If, however, we only observe communities smaller than *N*_max_, *Regime III* will not be observed, as we suspect is the case for the Wikipedia talk pages.

In summary, some aspects of the empirical patterns repeat across platforms, i.e., the *Regime I*-to-*II* phase transition and the existence of the third regime. With others, we exhibit higher variance across platforms, e.g., the slope of *Regime II* and the activity levels within *Regime III*. The full three-regime structure is observed in five of the seven platforms: TAP, BRDS, YOUT, RED, and HI5. In the other two platforms, WIKI and GOODR, we only observe two regimes. We speculate that these differences stem from differing platform contexts and sampling constraints. To also test whether the existence of the three regimes depends on community lifetime stage in other platforms, Figs I and J in S1 File are reconstructions of Fig 2 for YOUT and BRDS. They too show that the existence of the three-regime pattern does not depend much on community life-cycle stage, across platforms. We hope that future research will explore the effect of platform context on the activity–size relationship further.

## The model

### Online communities as a collection of branching discussion trees

We use a branching process model [26] to explain the observed activity–size patterns in which the three-regime structure is exhibited. A variety of generative models of online discussions have been suggested in the past. Some of these models assume that a preferential-attachment like growth is at the base of the growth of discussion trees. Another common factor in these works is the “age” of a message on the discussion tree. The longer the time that has passed since a message is posted, the lower the probability that it will receive responses. Time could either be discrete [24] or continuous [27]. Other works also refine and put additional behavioral traits to capture more realistic aspects of real-life discussion trees. For example, the root-bias: the tendency of the root post to attract more responses than its leaves [28] or reciprocity between users [29]. This behavioral trait is well-established in the digital world. Further works also model social influence and the effect of various roles of users (for a comprehensive review, see [30]).

Here, our focus is on theorizing and modeling the three-regime structure that naturally arises when users interact and generate joint discussion trees. Our unit of analysis is the community of users. In our model, a community consists of N interacting members that generate trees of messages and their responses.

We denote by *q*_*i*,*j*,*k*_ the rate of *i*’s response to user *j* for a given message *k*. In the interest of simplicity, we assume that, both *q*_*i*,*j*,*k*_ and *N* are time-, user- and message-independent. In that sense, we assume an effective constant response rate, *q*, per online community. A seed message is in fact a tree composed of only one message that may later evolve, or branch, into a tree of more than one message. In circumstances where a more complex model is needed, the responsiveness of a community would be represented by a matrix of response rates between all members, *Q* = *q*_*i*,*j*_, but, here we assume that it is a single homogeneous scalar *q*. We also allow the rate of response to be dependent on the depth of the tree. For example, it is natural to assume that discussions saturate, i.e., that the deeper the message within the tree, the lower the probability is that it will receive a response. We, therefore, will assume that the effective rate of response also depends on tree depth, *g*, in the following way: $q=\tilde{q}\cdot f(g)$, where *f*(*g*) is a general function of depth and $\tilde{q}$ is the depth-independent constant rate of response.

The evolution of message trees is modeled here as a Galton-Watson branching process. Let *Y*_*k*_ be an i.i.d. random variable representing the number of replies that post *k* receives. The *offspring distribution* Φ(*Y* = *κ*) is the distribution of these replies, and we assume it to be homogeneous across users and time. In our scenario, Φ(*κ*) depends only on *N* and *q*, i.e., Φ(*κ*) = Φ(*κ*|*N*, *q*). The general expression for the total number of messages, *Z*_*g*,*m*_, at tree depth *g* in tree *m*, is given by the following iterative relation:

$$\begin{array}{c} Z_{g,m}=\sum_{i=1}^{Z_{g-1,m}}Y_{i} \end{array}$$ (1)

Where *Z*_0,*m*_ = 1 initially because discussion trees initiate with one message. Now, we denote by Γ = Γ(*s*, *g*_max_|*N*, *q*, Φ(*κ*)) the probability to observe a tree of size *s* and maximal depth *g*_max_.

Finally, the mean community activity is the total activity in a community, scaled to community size. Assume the number of seed messages per user (i.e., the initial seed of a discussion tree) is a constant *p*. Note that *p* does not have to be discrete. It simply denotes the expected number, per person, of seeds. Now, the total activity of users in a community is the sum over the realizations of the tree sizes, i.e., the random variable *γ*_*s*_, drawn from the distribution Γ, divided by community size:

$$\begin{array}{c} A_{pc}=\frac{1}{N}\sum_{l=1}^{N\cdot p}\gamma_{s,i} \end{array}$$ (2)

Here, *p* is assumed to be homogeneous across time and users. An analytic solution to (2) is intractable for our relevant case [31] for the same reasons that apply to Eq (1). Notably, in our case, the response rate depends on the depth of the tree, $q=\tilde{q}\cdot f\left(g\right)$. This further complicates the ability to find analytic solutions. Therefore, in order to fit the data to our model and to see whether the model replicates the observed three-regime structure, we use numerical simulations and maximum likelihood estimations (see below). In what follows, we show how the model exhibits a phase transition behavior and that this behavior can be explained in the context of online communities.

### Phase transition of discussion trees

In order to gain insight into how tree growth depends on community size, we denote the mean number of replies as the expectation of the offspring distribution, 〈Φ(*κ*)〉 ≡ *μ*(*N*, *q*). Using (1), we write the expected number of replies per message *M*_*g*_ at depth, or generation *g*, as the following recursive relationship:

$$\begin{array}{c} \left\langle Z_{g}\right\rangle \equiv M_{g}=\mu \left(N,q\right)\cdot M_{g-1} \end{array}$$ (3)

Here, for the purpose of exploring the basic phase transition property of discussion trees, we assume that *f*(*g*) = 1, i.e., *q* is independent of depth. In a more general scenario, one can take the dependency of *q* on *g* through *f*(*g*) into consideration, when calculating the expectation *μ*. Under this assumption, and due to the fact that *M*_0_ = 1, we arrive at the closed expression:

$$\begin{array}{c} M_{g}=\mu {\left(N,q\right)}^{g} \end{array}$$ (4)

Eq (4) demonstrates that the mean size of the tree is “geometrically sensitive” to the first moment of the distribution of replies, *μ*(*N*, *q*). The critical point of growth occurs for *μ*(*N*, *q*) = 1. If *μ*(*N*, *q*) > 1, a super-critical branching process is in effect, and so tree posts will geometrically grow across generations. On the other hand, in the sub-critical case, *μ*(*N*, *q*) < 1, the expected number of replies shrinks geometrically. This is a known property of branching trees [26].

### An approximation for the critical community size

To interpret the physical meaning of the critical point of activity growth in the context of online communities, we use (4) to explicitly write the condition for critical growth:

$$\begin{array}{c} \mu (N_{\text{crit}},q)=1 \end{array}$$ (5)

*N*_crit_ is the critical size of a community in which discussion trees theoretically “explode.” It can be calculated using Eq (5). We assume here, again, that the rate of responsiveness, *q*, is constant and independent of users, time, or tree depth. In this case, the expression for *N*_crit_ is then simply:

$$\begin{array}{c} N_{\text{crit}}=\frac{1}{q} \end{array}$$ (6)

Essentially, (6) shows that the critical size of a community is determined, in this simple scenario, by the level of responsiveness. The higher the inherent responsiveness of the community is, the lower the critical threshold.

### Estimating the model

We use a Maximum Likelihood Estimator (MLE) to test several configurations of our model and find the one that shows the best fit to the data. We use the TAP data, where we have the best user- and message-level resolution (see S1 File for complete details). Table 1 lists the four models that we test. The MLE, in practice, renders a statistical estimation of the offspring distribution parameters Φ(*κ*|*N*, *Q*(*g*)), namely, the responsiveness paramater, *q*, *N*_max_, and a third parameter, λ, that is used to model the decline of response probability with increasing tree depth. For simplicity, we choose the Binomial distribution as the basis of the offspring distribution. For robustness, in S1 File, we also present estimations using two other distributions: Poisson and Negative Binomial. These estimations show that the results of the Binomial distribution are mostly replicative (see S1 File for details). Per each observation, in order to estimate the parameters, we use the observed number of offspring and the number of users that were active, *N*. To reduce noise, in each observation, we count the number of users active in the community within a time window of three months, centered around the observation time. The results are qualitatively similar for a variety of time windows choices. We test four configurations of our model, from a simple single-parameter configuration to a full-featured three-parameter one. Model 1 estimates only the responsiveness, *q*, i.e., a model that does not include a third regime or tree-depth dependent decay. Model 2, on the other hand, incorporates the third regime, but not the tree-depth decay. Finally, Models 3 and 4 include both the third regime and the tree-depth decay. The difference between Models 3 and 4 is the functional form of the tree-depth decay, namely, an exponential decay or power-law decay, respectively.

**Table 1:** Discussion trees model types.

Model name	Decay type	Third regime (*N*_max_)	Response rate (*q*)
Model 1	None	No	Yes
Model 2	None	Yes	Yes
Model 3	*f*(*g*) = *e*^−*g*/λ^ (Exponential)	Yes	Yes
Model 4	*f*(*g*) = *g*^−λ^ (Power law)	Yes	Yes

For robustness, we also estimated the set of models for two more random samples (see Tables A and B in S1 File). Interestingly, the model showing the lowest information criterion is Model 4, our full model, which includes a power law decay of the probability of response. Model 4 was the best fit model also across all our robustness checks (see S1 File). Exponential decay seems to produce a worse fit, across the board, also supported by an instability of parameter values in the robustness checks. Adding the third regime (*N*_max_) in the model increases the fit considerably. Including the tree-depth decay in the model (λ) also increases the fit.

**Fig 6:**
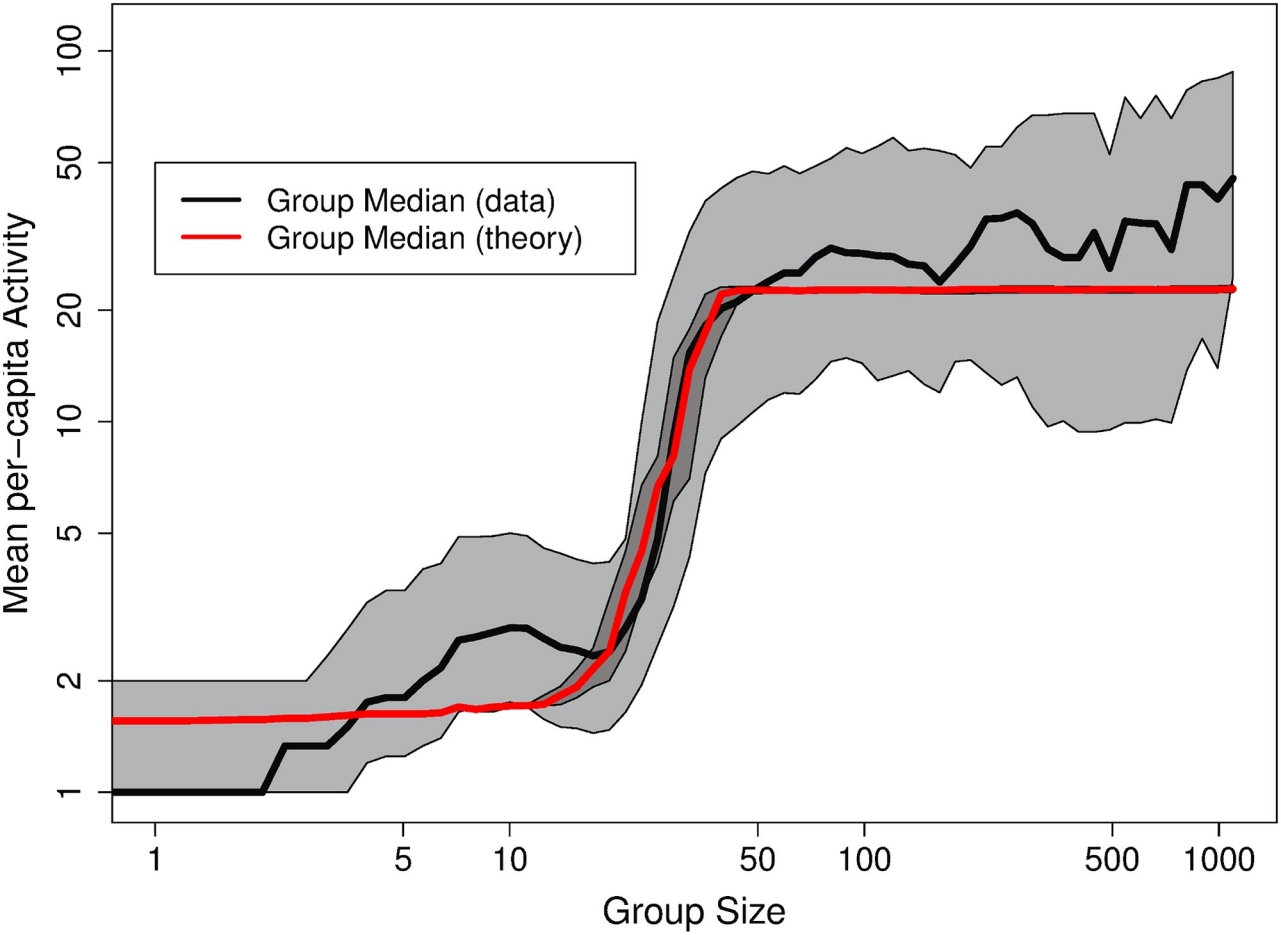
Fit of MLE estimations. The model fit is shown in red and the respective percentile envelope overlay the data, as in 1.

The For clarity, a visualization of the fit of Model 4 is shown in Fig 6. The fit is the result of simulations of 2, 000 discussion trees per group size, according to the model [Eq (2)] and using parameter values from Table 2. The red curve and shaded area are calculated the same way as they are for Fig 1. Notably, this model exhibits the same three regimes and the sharp *Regime I*–*II* transition. Note that the curve is within the 25%–75% percentile envelope of the observations. Interestingly, the estimated responsiveness $\tilde{q}=0.033$ is close to our initial, crude, estimations and corresponds to *N*_crit_ ≈ 30. Finally, another interesting observation is that given the estimated value of λ, the functional form of the rate of deceleration of tree growth, *f*(*g*), is close to a one-over-square-root form: $\frac{1}{\sqrt{g}}$.

**Table 2:** MLE estimations.

Parameter	Model 1	Model 2	Model 3	Model 4
$\tilde{q}$	0.003*** (2.5e-05)	0.032*** (3.3e-04)	0.031*** (3.5e-04)	0.038*** (5.3e-04)
*N*_max_	−−	26.2*** (0.011)	31.4*** (0.266)	41.4*** (0.221)
λ	−−	−−	27.7*** (1.187)	0.62*** (0.013)
Akaike IC	56,332	55,096	55,137	52,601
Likelihood Ratio				
Test (*χ*^2^)	32***	46***	84***	2,112***
Observations	20,000	20,000	20,000	20,000

***p<0.01,

**p<0.05

*p<0.10

Results of the Maximum Likelihood estimations of four potential models of the discussion trees, for the TAP dataset. Likelihood ratio tests were conducted for each model vs. the intercept, no-predictors model. (std. errors in parentheses).

To test whether the goodness of fit replicates with the other data sets, we conducted similar estimations for the rest of our data sets. The full detail of the procedure and the results for each data set is given in S1 File. In general, the fit to Model 4 (Table 1) is signifiin cantly better than the benchmark. Notably, while the model fits TAPUZ, YOUT, BRDS, GOODR and HI5 quite well (see S1 File), it does provide a lesser fit to both the WIKI and RED data sets. Still, we argue that these findings establish the fact that even though the phase-transition patterns are not strictly universal, the model is useful to account for the activity-size patterns across most of the platforms we investigated.

## Conclusions

Our findings provide insight into the factors that underlie the emergence and sustainability of online communities. We find that the relationship between activity levels and size in these communities exhibits a three-regime pattern that mostly repeats across platforms and time, with exceptions. Further, we observe a sharp transition between two of the regimes and evidence for the existence of a critical community size. Below that critical size, member activity is largely uncorrelated, and so activity remains low and sporadic. Above that critical size, member activity becomes increasingly correlated, and an interactive community emerges. We argue that the regimes’ structure and this sharp transition can be explained by a dynamic model of peer-to-peer communications that generate trees of interactions. The model explains the sharp transition as the result of the multiplicative nature of the interaction trees. Above a certain member-to-member reply rate, trees grow geometrically. In the context of online communities, the geometric growth results from an interplay between community size and the ambient level of responsiveness. The characteristic scale of the critical point of growth, is determined by the level of member-to-member responsiveness in a community. The higher the responsiveness, the lower the critical point which defines the size above which interactions boom in the community.

A limitation of our findings is that we only observe correlations and are not able, in this non-experimental context, to demonstrate that size actually causes the transition between regimes. Having said that, we find that the model fits the data well (e.g., in Fig 6), in spite of its relative simplicity, including the observed regimes and the sharp transition patterns. Further, throughout the paper, we present indirect and corroborating evidence for the suggested theory. Future work could investigate more complex forms of the model, such as we reviewed in the model section, and the implications of some of our simplifying assumptions. Another limitation is that we do not model the differences across platforms which are, presumably, the underlying reasons for the differences in empirical patterns. The question of which specific difference between platforms can explain the the empirical observation differences is an opportunity for more further research. Furthermore, our paper contributes to the computational social science literature. While sharp phase transitions in social systems were hypothesized, mainly by theory [23], the empirical evidence to support these conceptualizations was, so far, lacking. Here, we are among the first to present direct evidence for sharp transitions of collective social behavior. Finally, it is known that within communities, there exists heterogeneity of contribution [5]. This heterogeneity most likely affects the propensity of a community to thrive or fail. Further research should empirically investigate the sources and outcomes of contribution heterogeneity within online communities.

## Materials and methods

Online discussion groups are constantly being created and maintained by members in designated online platforms. Generally, a discussion topic initiates with a single message posted by a user on the platform. Other users can post replies to that message or to the following messages such that a tree of posts and replies develops. We collected time-stamped group discussion comments at random from context-free platforms such as Tapuz, Goodreads, hi5.com, boards.ie, YouTube, and focal group websites like the Wikipedia article talk pages or the technologically oriented Reddit. For each dataset:

1. Data were scraped from publicly available online web pages or online services.
2. Datasets included a time stamp per each comment/post that was posted on the respective platform, the unique user id of the user who posted it, the specific community that the comment was posted on, and where available which comment the focal comment replied to.
3. In some cases, if the exact network of posts and replies was required, the records were processed to locate parent–child (directional) links between pairs of comments. In some platforms (e.g., Tapuz), the structure of a discussion page is such that users can choose to respond to a certain post and create a clear thread where each “child” is directly connected to their “parent” response. In other platforms, the child–parent relationship is approximated by either marking the immediate following message, or better still, a user from a preceding comment (the parent comment) may be referenced using hash symbols, similar to the re-tweet mechanism in Twitter. Some platforms (e.g., MediaWiki) may further convert these name mentions to user-page links.

The full data then include the time–stamped records of posts and replies that collectively thread into a tree graph of discussions that has measurable depth (maximal thread path length), volume (number of comments), breadth (number of leaves), community size (number of unique participants), and activity level (number of comments per time unit).

In terms of ensuring that data sampling methods are consistent across platforms, in order to rule out sample selection and to create consistent data formats, our general rule was the following; We sampled from each platform community either by collection of all complete activity within a given and lengthy (several years) period time slice where possible, or when collecting the complete data was prohibitive, to collect a large sample of communities at random. Per each community, of course, all activity was collected up to the time of collection. For example, for the Tapuz data (www.tapuz.co.il/communa), we collected all “communes” (user-generated discussion forums) that were active between the years 2004 and 2016. For the case of Wikipedia, we used the “random page” function in MediaWiki to sample 21,000 pages. In hi5.com, all the discussion topics from 2009 to 2016 were collected. Similarly, we downloaded YouTube video page data using a third-party tool (www.npmjs.com/package/youtube-random-video and tools.digitalmethods.net/netvizz/youtube/mod_video_info.php) to sample 10,000 videos at random. Table 3 provides a descriptive summary of the collected data. The empirical analyses performed on the data are explained in detail within the text or in S1 File (e.g., the Maximum Likelihood Estimation).

**Table 3:** Summary statistics of the datasets that were used.

Sample	Ngroups	Nusers	Nposts	Age[yr]
HI5	126,468	330,936	2,568,352	7
TAP	10,122	134,747	9,986,206	12
RED	14,869	764,562	10,000,000	8
GOODR	8,040	42,345	171,411	7
WIKI	17,969	30,506	154,469	15
BRDS	624,083	57,796	1,870,566	15
YOUT	8,446	1,104,906	1,567,073	10

HI5 represents data from hi5.com, TAP comes from www.tapuz.co.il/forums, RED are reddit posts from reddit.com/r/datasets/comments/3mg812, GOODR data are from Goodreads www.goodreads.com, WIKI are Wikipedia talk pages, BRDS are www.boards.ie, and YOUT are user comments on YouTube video pages.

## Supporting information

S1 File Supporting information file. Contains More patterns of activity vs. size, discussions on the dependence of discussion tree growth on its depth, the maximum likelihood estimations and fit (description of the procedure), and robustness checks of the MLE.
(PDF)
