# Supplementary material for: Emergence of online communities: Empirical evidence and theory
Source: PLoS One. 2018 Nov 14;13(11):e0205167. doi: 10.1371/journal.pone.0205167 (PMC6333374; doi:10.1371/journal.pone.0205167)
Supplement: S1 File — Contains More patterns of activity vs. size, discussions on the dependence of discussion tree growth on its depth, the maximum likelihood estimations and fit (description of the procedure), and robustness checks of the MLE. (PDF) [file pone.0205167.s001.pdf]

Supporting Information for: Emergence of online communities: Empirical evidence and theory

Dover and Kelman

Supporting Information (SI)

a. More patterns of activity vs. size

Here we show the per-capita activity vs. size relationship for two additional platforms. A Fig. shows the median per-capita activity for the HI5 dataset. The expected three regimes clearly exist in the curve. Unlike the TAP or BRDS case, the third regime occurs at relatively low mean activity levels. We suspect that the HI5 platform does not encourage longer, chain-like conversations, as, e.g., the TAP and BRDS platforms do. In other words, while discussion trees undergo the sharp transition from Regime I to II, the probability of response decays steeply with tree depth, probably because of the design and context of usage of the platform. The curve in B Fig. (RED) also exhibits the expected three regimes, with noticeable discrepancies. The first regime occurs only for single-user communities. In that sense, it is similar to the GOODR set. The interpretation here, as it is for GOODR, is that the prevalent responsiveness is already high for micro communities. Further, it seems that the transition into the third regime occurs across a relatively wide range of community sizes. Although out of scope in this paper, we can speculate that this could be the outcome of heterogeneity across communities.

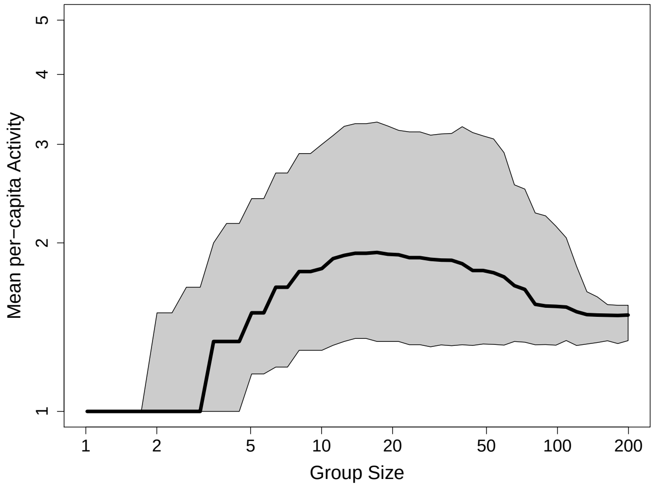

Figure A. Median group mean activity across a range of community sizes in the HI5 data. The median activity is the solid thick line, the shaded areas mark the regions between the 25th to the 75th percentiles.

b. The dependence of discussion tree growth on depth

We assume, in the model, that the growth of discussion trees, e.g., in equation (2), depends on tree depth. Essentially, this assumption takes into account that as a discussion

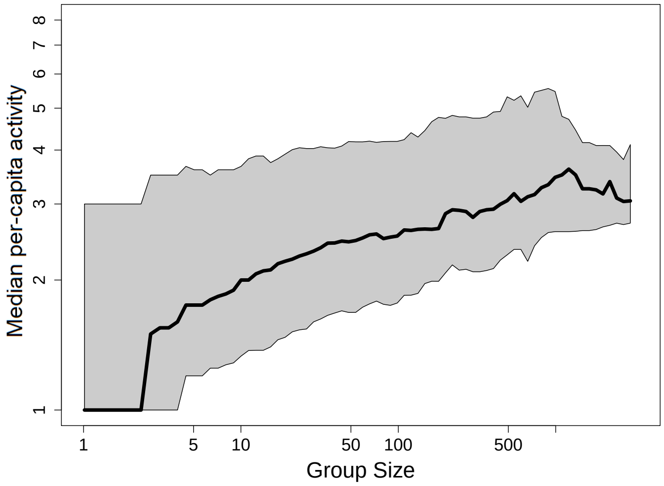

Figure B. Median group mean activity across a range of community sizes in the RED data. The median activity is the solid thick line, the shaded areas mark the regions between the 25th to the 75th percentiles.

tree grows longer and deeper, the probability of response to higher-depth messages, decays. One way to model this is by allowing the responsiveness to depend on generation, g, i.e., $Q = Q(g)$.

There are several functional forms that can represent this tendency of the response probability to decrease with tree depth that cannot be ruled out, theoretically. With the MLE procedure, we tried several functional forms. Here, we report the two forms that provide the highest level of fit; The exponential decay form: $Q(g) = C \cdot e^{-\lambda \cdot g}$ and the power law decay form: $Q(g) = C \cdot g^{-\lambda}$. The best fit, by far, occurred when we used the power law decay form. Therefore, we use the power law form in the estimation reported in the main part of the paper. Interestingly, the MLE estimation of the power law form, exhibit a value of $\lambda \cong \frac{1}{2}$. In other words, our estimation shows that it seems that the functional form of the rate of deceleration of tree growth, $f(g)$, closely approximates $g^{-\frac{1}{2}}$.

c. Maximum likelihood estimations and fit - description of the procedure

The purpose of the MLE process is to estimate the parameters of the offspring distribution function $\Phi\big(\kappa \big| N, Q(g)\big)$. Notably, the full dynamic picture of discussion tree growth is captured in the offspring distribution function. In our basic scenario, the distribution is homogeneous across tree nodes and solely depends on four parameters: N, $N_{\max}$, q and g. These are the size of the community, the maximal size of community interaction, the constant community responsiveness and the depth of the node in a discussion tree, respectively. In our case, N is known per community, per node and g is known, per node. The observed random variable, i.e., the number of replies per node, κ, is also observed. Under the assumptions of the model, the offspring distribution is taken to be binomial. Therefore, the likelihood function can be written as:

$$\mathcal{L}(q, N_{\max}, \lambda) = \prod_{i,j,k} B\Big(\mathcal{N}(N_i, N_{\max}),\ q \cdot f(\lambda, g_{i,j,k})\Big) \qquad (1)$$

The indices i, j, k are for community, tree and node, respectively. The sample size of

the binomial process is $\mathcal{N}(N_i, N_{\max}) = \min\{N_i, N_{\max}\}$, i.e., either community i's size, N_i if $N < N_{max}$ or N_{max} otherwise. The community responsiveness decays with tree depth g. The functional form of this decay is expressed by $f(g)$ as explained in the previous section. Here, we write the decay function as $f(\lambda, g_{i,j,k})$ to denote that the depth varies across trees and nodes. Maximum likelihood is obtained by the bounded Broyden-Fletcher-Goldfarb-Shanno (BFGS) approach. The solution is global as we use random initial conditions across a wide range of parameters and runs. The results of the estimation are given in Table 2 in the main paper.

To achieve the fit shown in Fig 6, we used the following process. Using the MLE parameters, we simulated trees as a function of community size. For each community, we simulated 1000 trees and for each community size we created 1000 simulated communities. The chosen numbers of trees and community sizes did not have a qualitative effect on the results. Using the MLE and model-based simulated communities, we calculated the median per-capita activity as a function of size. The result is shown in Fig 6.

d. Maximum likelihood estimations - Robustness

d..1 More samples

To gauge the robustness of the MLE estimations, which were done on a sample of $20,000$ observations, we estimated two more random samples. The results are presented in A and B Tables. The additional two samples' results show that the estimations for our main model (4), are stable across samples, as are the results for model 2. On the other hand, estimations for model 3 and 1 are less stable. We interpret this as a hint that the power-law decay function may be a more-appropriate choice to capture discussion-tree dynamics.

Parameter	Model 1	Model 2	Model 3	Model 4
$\tilde{q}$	0.003***	0.034***	0.028***	0.037***
	(2.6e-05)	(3.5e-04)	(2.7e-04)	(5.3e-04)
$N_{\max}$	−−	23.8***	35.9***	42.5***
		(0.075)	(0.266)	(0.234)
λ	−−	−−	21.4***	0.65***
			(0.697)	(0.014)
Akaike IC	56,423	55,112	55,308	52,605
Likelihood Ratio				
Test (χ^2)	28***	62***	255***	2,396***
Observation	20,000	20,000	20,000	20,000

***p<0.01, **p<0.05 *p<0.10

Table A. MLE estimations - second sample. Results of the Maximum Likelihood estimations of four potential models of the discussion trees, for the TAP dataset. (Standard errors in parentheses)

d..2 Other base distributions

The naive assumption of our base model is that the basic form of the offspring distribution is binomial. To test whether this assumption is a simplification of the empirical reality, we test two other distributions: the Poisson distribution and the Negative Binomial distribution. In the Binomial scenario the rate of replies (offspring) is assumed

Parameter	Model 1	Model 2	Model 3	Model 4
$\tilde{q}$	0.014***	0.032***	0.026***	0.037***
	(1.0e-04)	(2.7e-04)	(2.1e-04)	(5.2e-04)
$N_{\max}$	−−	28.2***	64.1***	43.5***
		(0.070)	(1.737)	(0.097)
λ	−−	−−	13.8***	0.36***
			(0.295)	(0.010)
Akaike IC	48,601	48,290	49,760	47,818
Likelihood Ratio				
Test (χ^2)	112***	146***	1321***	620***
Observation	20,000	20,000	20,000	20,000

***p<0.01, **p<0.05 *p<0.10

Table B. MLE estimations - third sample. Results of the Maximum Likelihood estimations of four potential models of the discussion trees, for the TAP dataset. (Standard errors in parentheses)

to be the result of N people, each supposedly choosing at random whether to reply or not, with some probability q. The Poisson process, on the other hand, assumes that there is a set mean of replies and a random process in a fixed time window. Last, in the Negative Binomial scenario a different choice of process is made. The Negative Binomial distribution allows a more dispersed distribution of offspring. The results of the two latter forms are presented in C and D Tables. We use the same sample from the main draft for these estimations, containing $20,000$ observations.

Parameter	Model 1	Model 2	Model 3	Model 4
$\tilde{q}$	0.003***	0.075***	0.026***	0.040***
	(2.4e-05)	(6.2e-04)	(2.5e-04)	(5.5e-04)
$N_{\max}$	−−	10.8***	42.9***	42.1***
		(0.409)	(1.112)	(0.133)
λ	−−	−−	14.1***	0.70***
			(0.334)	(0.014)
Akaike IC	54,478	54,479	55,420	52,329
Likelihood Ratio				
Test (χ^2)	5***	5***	933***	2157***
Observation	20,000	20,000	20,000	20,000

***p<0.01, **p<0.05 *p<0.10

Table C. MLE estimations - Poisson distribution. Results of the Maximum Likelihood estimations of four potential models of the discussion trees, for the TAP dataset. The base distribution for the offspring distribution here is a Poisson distribution. (Standard errors in parentheses)

Both C and D Tables show that the evaluation of the basic probability of reply is more-or-less robust. In D Table, the base distribution is Negative Binomial and thus the interpretation of the probability should be one minus the estimated q. The events

(which in our data are replies) are considered ‘failures’ in the language of the negative binomial distribution. The decay of offspring rate with tree depth is captured in the Poisson scenario, but not as much in the Negative Binomial scenario. This suggests that the Negative Binomial may not be a good choice of base distribution.

The overall impression is that both distributions capture somewhat more variance, in the sense that the they lower the AIC in model 4. We recognize that the real-life base probability distribution may be more complex than a simple binomial distribution and we hope to explore and address this in future research.

**Parameter**	**Model 1**	**Model 2**	**Model 3**	**Model 4**
$\tilde{q}$	0.931***	0.968***	0.947***	0.949***
	(4.5e-04)	(3.1e-04)	(7.4e-04)	(1.1e-03)
$N_{\max}$	−−	28.5***	10.5***	16.8***
		(0.084)	(0.019)	(0.132)
λ	−−	−−	174.6***	0.009***
			(3.121)	(7.5e-04)
Akaike IC	141,636	48,403	50,884	48,253
Likelihood Ratio				
Test (χ^2)	93,331***	76***	2555***	76***
Observation	20,000	20,000	20,000	20,000

***p<0.01, **p<0.05 *p<0.10

Table D. MLE estimations - Negative Binomial distribution. Results of the Maximum Likelihood estimations of four potential models of the discussion trees, for the TAP dataset. The base distribution for the offspring distribution here is a Negative Binomial distribution. (Standard errors in parentheses)

d..3 MLE estimations for other datasets

E Table shows the estimated parameters for the rest of the datasets. Notably, unlike the TAP dataset, we do not observe the structure of the trees. This is because these platforms do not explicitly render a user’s answer to a specific message, but rather they generally allow a user to “chain” their message in a thread of messages.[1] One exception is YouTube that allows a response, but only to a depth of one level within the tree. With YouTube, there is no structure beyond the second level of the tree. Users have learned to use tags in the response body in order to denote who they refer to. However, we cannot be sure that the references are complete. Therefore, we used a different MLE process where we fit the model to the data on the group level rather than on the individual message level. Per each set of parameters ($\tilde{q}$, $N_{\max}$ and probability of tree appearance, p), we simulated an ensemble of trees to generate simulated groups. Given the lower level of this data, we cannot identify λ. We then fit, using MLE, the median of the data to the median of the simulated groups, conditional on the parameters. The results are given in E Table and visualized in C to H Figs. The goodness of fit was measured using Welch t-test between the benchmark model and the main model’s distribution of Mean Square Errors. With the exclusion of the RED and WIKI datasets, the model fit looks adequate and seems to provide significantly better fit than the benchmark model (which in this case, we took to be just the intercept as q). Although we cannot explain

[1] An exception to this is the RED platform which does allow linking replies to specific origin posts. The version of the public dataset we acquired does not include these data.

the difference between these two data (RED and WIKI) and the rest, we note that even though the model shows that the exact pattern is not universal, it seems to be useful across platforms.

Table E. MLE estimations for other datasets

Parameter	YOUT	BRDS	WIKI	GOODR	HI5	RED
$\tilde{q}$	0.22***	0.013***	0.019***	0.20***	0.14***	0.24***
	(0.068)	(1.7e-04)	(3.3e-04)	(0.023)	(1.3e-03)	(7.2e-03)
$N_{\max}$	5.04***	179.8***	47.7***	7.1***	6.5***	5.2***
	(0.221)	(0.657)	(0.566)	(0.524)	(0.936)	(0.524)
p	0.21***	0.36***	1.06***	1.0***	0.47***	031***
	(0.007)	(0.069)	(0.012)	(0.015)	(0.021)	(0.049)
Welch T-Test for MSE Difference	6.3***	4.2***	4.15***	4.75***	5.11***	6.46***
Observations	20,000	20,000	20,000	20,000	20,000	20,000

***p<0.01, **p<0.05 *p<0.10

Results of the Maximum Likelihood estimations of Model 4 for YOUT, BRDS, WIKI, GOODR. HI5, and RED datasets. Welch t-tests were conducted vs. the benchmark intercept-only model. (std. errors in parentheses).

d..4 Activity-size for communities of varying life-cycle stages - Youtube and iBoards

I and J Figs illustrate the activity-size curves for two more platforms across communities life-cycle stages. These are similar to the life-cycle stages curve shown of the TAP dataset in the main paper (Fig 2) but for other platforms. Here, again, communities were divided into groups of different age (i.e., time since initiation) and, per each group, the activity-size curve was calculated and plotted. Similar to the TAP case, we observe that the three-regime pattern exists throughout the communities lifetime. Unlike the TAP case, it is hard to interpret the differences of the activity-size curves between age tiers. We note in passing that since the transition in the BRDS case (J Fig) occurs for relatively large community sizes (> 50), the samples for the curve calculation in the large-sizes end are small. The implications are: **(1)** instead of four categories of age we have three in J Fig, and **(2)** the sample sizes for very large communities (around > 300) were insufficient and so the curve ends at lower sizes compared to Fig 5(a).

References

[1] Zhang J, Ackerman MS, Adamic L. Expertise networks in online communities: Structure and algorithms. In: Proceedings of the 16th International Conference on World Wide Web. ACM; 2007. p. 221–230.

[2] Kozinets RV. The field behind the screen: Using netnography for marketing research in online communities. J Marketing research. 2002;39(1):61–72.

[3] González-Bailón S, Borge-Holthoefer J, Rivero A, Moreno Y. The dynamics of protest recruitment through an online network. Sci Rep. 2011;1:197.

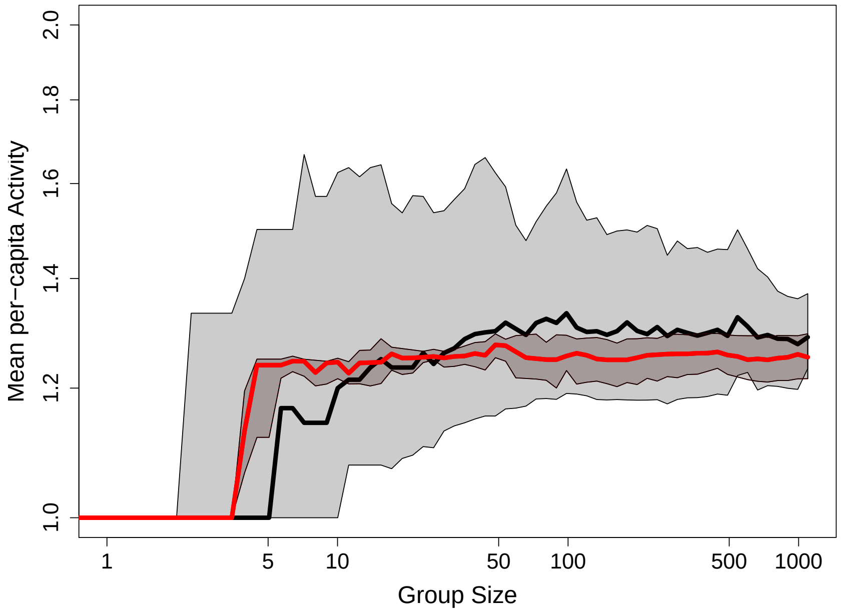

Figure C. Fit of MLE estimations for YOUT. The model fit is shown in red and the respective percentile envelope overlay the data, as in Fig 1.

[4] Grabowicz PA, Ramasco JJ, Moro E, Pujol JM, Eguiluz VM. Social features of online networks: The strength of intermediary ties in online social media. PloS ONE. 2012;7(1):e29358.

[5] Oliver P, Marwell G, Teixeira R. A theory of the critical mass. I. Interdependence, group heterogeneity, and the production of collective action. American J Sociology. 1985; p. 522–556.

[6] Olson M. The logic of collective action: Public goods and the theory of groups. vol. 124 of Harvard Economic Studies. 20th ed. Cambridge Massachusettes, London England: Harvard University Press; 1971.

[7] Raban DR, Moldovan M, Jones Q. An empirical study of critical mass and online community survival. In: Proceedings of the 2010 ACM conference on Computer supported cooperative work. ACM; 2010. p. 71–80.

[8] Oliver PE, Marwell G. Whatever happened to critical mass theory? A retrospective and assessment. Sociological Theory. 2001;19(3):292–311.

[9] Dabbish L, Farzan R, Kraut R, Postmes T. Fresh faces in the crowd: Turnover, identity, and commitment in online groups. In: Proceedings of the ACM 2012 Conference on Computer Supported Cooperative Work. ACM; 2012. p. 245–248.

[10] Kleineberg KK, Boguná M. Digital ecology: Coexistence and domination among interacting networks. Sci Rep. 2015;5:10268.

[11] Dover Y, Kelman G. Nucleation of social groups: The role of centrality inequality and social mobility. In: Cherifi C, Cherifi H, Karsai M, Musolesi M, editors. Complex Networks & Their Applications VI. The Sixth International Conference on Complex Networks and Their Applications. vol. 689. Springer International Publishing; 2017. p. XXV–1288.

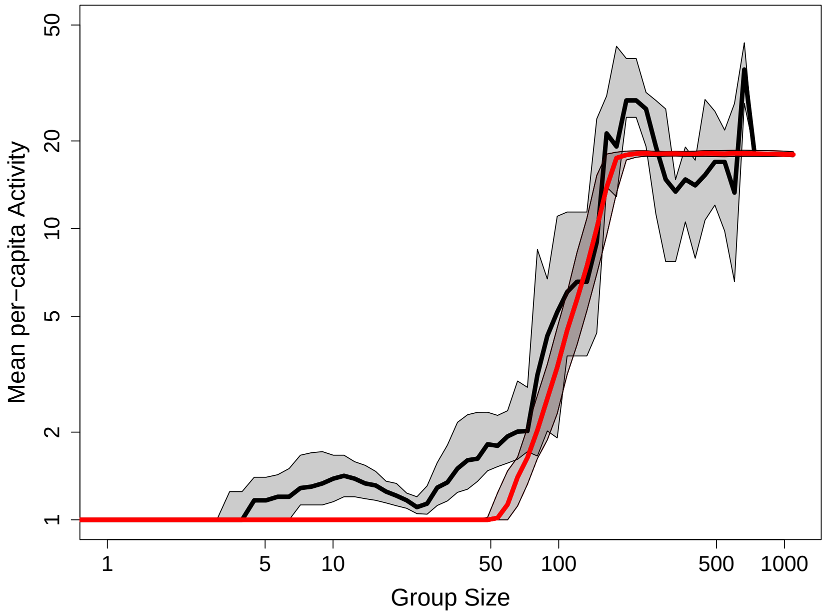

Figure D. Fit of MLE estimations for BRDS. The model fit is shown in red and the respective percentile envelope overlay the data, as in Fig 1.

[12] Han X, Cao S, Shen Z, Zhang B, Wang WX, Cressman R, et al. Emergence of communities and diversity in social networks. Proceedings of the National Academy of Sciences. 2017;114(11):2887–2891.

[13] Bishop J. Increasing participation in online communities: A framework for human–computer interaction. Comp H B. 2007;23(4):1881–1893.

[14] Johnson SL, Butler B, Faraj S, Jarvenpaa SL, Kane GC, Kudaravalli S. New directions in online community research. In: International Conference on Information Systems Proceedings; 2010. p. 173.

[15] Ribeiro B, Faloutsos C. Modeling website popularity competition in the attention-activity marketplace. In: Proceedings of the Eighth ACM International Conference on Web Search and Data Mining. ACM; 2015. p. 389–398.

[16] Kleineberg KK, Boguñá M. Competition between global and local online social networks. Scientific reports. 2016;6:25116.

[17] Fortunato S. Community detection in graphs. Phys Rep. 2010;486(3-5):75–174.

[18] Preece J, Maloney-Krichmar D. Online communities: Design, theory, and practice. J Compute Mediat Comm. 2005;10(4):JCMC10410.

[19] Haythornthwaite C, Wellman B. Work, friendship, and media use for information exchange in a networked organization. J Am Soc Info Sci. 1998;49(12):1101–1114.

[20] Wellman B. Structural analysis: From method and metaphor to theory and substance. Cont Stu Soc. 1997;15:19–61.

[21] De Souza CS, Preece J. A framework for analyzing and understanding online communities. Interact Comput. 2004;16(3):579–610.

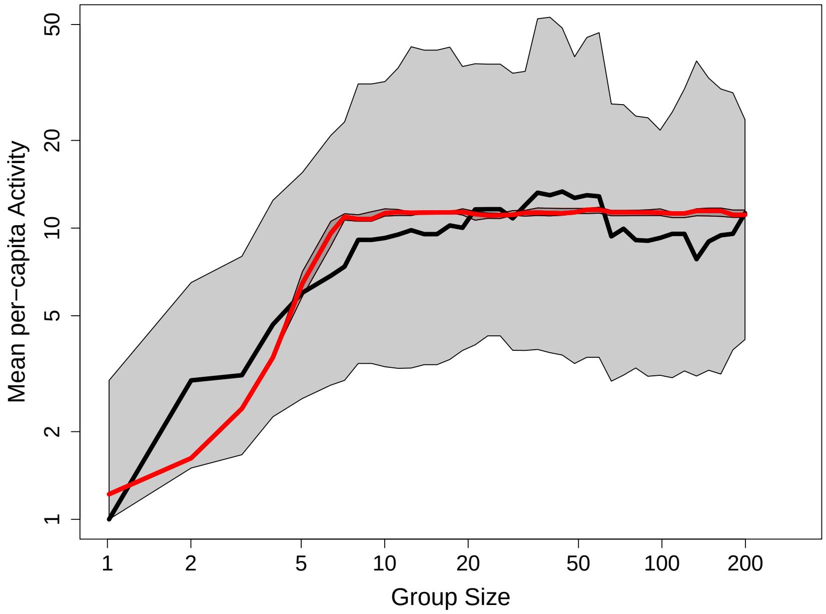

Figure E. Fit of MLE estimations for GOODR. The model fit is shown in red and the respective percentile envelope overlay the data, as in Fig 1.

[22] Preece J. Online communities: Researching sociability and usability in hard to reach populations. Australas J Info Sys. 2004;11(2).

[23] The definitions we cite consider communities to be separate and distinct groups of people. But, our data is secondary and extracted from the online world and, therefore, we have to make a simplifying assumption. Throughout the paper, we assume that when a group of people congregates around a common theme (e.g., within a topical discussion forum or around a piece of online content), they form for this purpose an ad-hoc community that is separate from other communities on the platform. We ignore, in that sense, the fact that within and across platforms, the membership of communities may overlap. We hope that future research will tackle this distinction and explore the consequences of making this assumption.;.

[24] Castellano C, Fortunato S, Loreto V. Statistical physics of social dynamics. Rev Mod Phys. 2009;81(2):591.

[25] Kumar R, Mahdian M, McGlohon M. Dynamics of conversations. In: Proceedings of the 16th ACM SIGKDD international conference on Knowledge discovery and data mining. ACM; 2010. p. 553–562.

[26] Voss J. Measuring wikipedia. In: 10th International Conference of the International Society for Scientometrics and Informetrics. Stockholm (Sweden); 2005. p. 24–28.

[27] Athreya KB, Jagers P. The IMA Volumes in mathematics and its applications. Classical and Modern Branching Processes Series. vol. 84. Springer-Verlag, New York, Berlin; 1997.

[28] Wang C, Ye M, Huberman BA. From user comments to on-line conversations. In: Proceedings of the 18th ACM SIGKDD international conference on Knowledge discovery and data mining. ACM; 2012. p. 244–252.

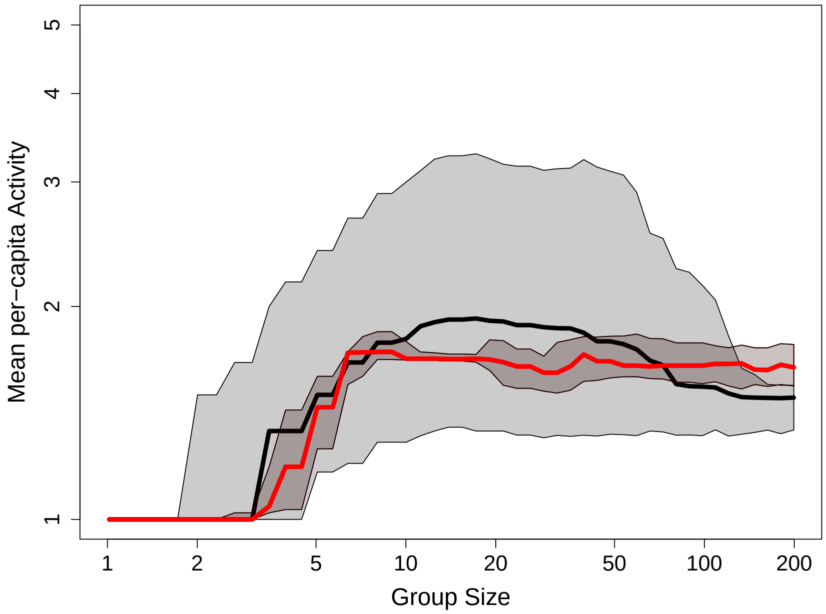

Figure F. Fit of MLE estimations for HI5. The model fit is shown in red and the respective percentile envelope overlay the data, as in Fig 1.

[29] Gómez V, Kappen HJ, Litvak N, Kaltenbrunner A. A likelihood-based framework for the analysis of discussion threads. World Wide Web. 2013;16(5-6):645–675.

[30] Aragón P, Gómez V, Kaltenbrunner A. To Thread or Not to Thread: The Impact of Conversation Threading on Online Discussion. In: The 11th International AAAI Conference On Web And Social Media (ICWSM-17); 2017. p. 12–21.

[31] Aragón P, Gómez V, García D, Kaltenbrunner A. Generative models of online discussion threads: state of the art and research challenges. Journal of Internet Services and Applications. 2017;8(1):15. doi:10.1186/s13174-017-0066-z.

[32] Corral A, Font-Clos F. Criticality and self-organization in branching processes: Application to natural hazards. arXiv preprint arXiv:12072589. 2012;.

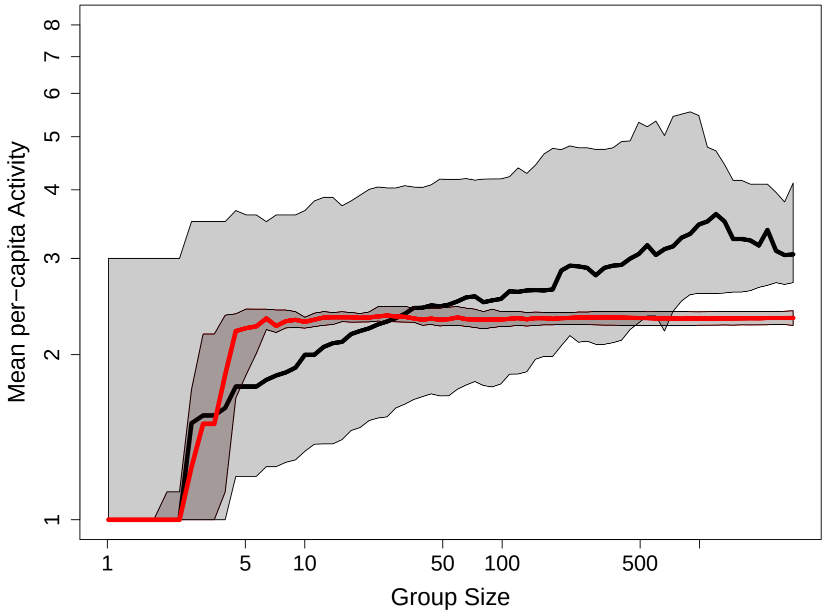

Figure G. Fit of MLE estimations for RED. The model fit is shown in red and the respective percentile envelope overlay the data, as in Fig 1.

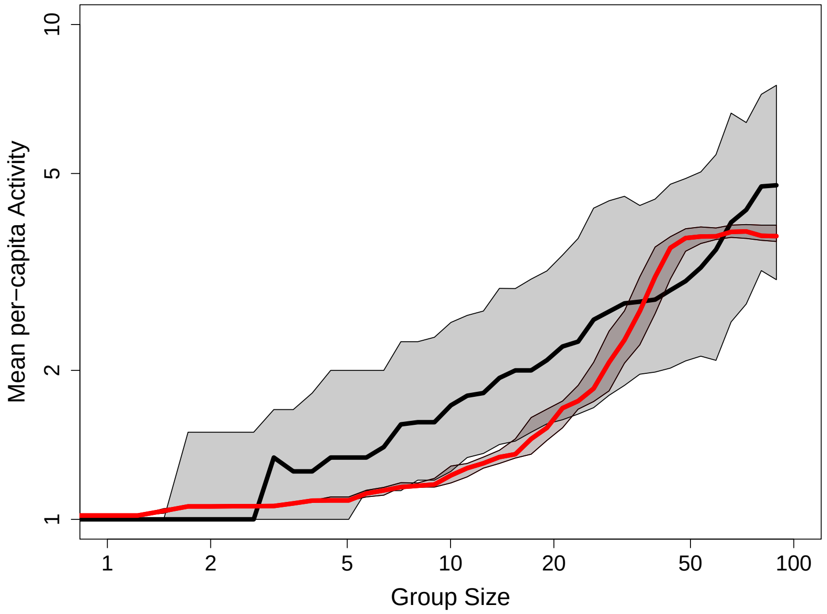

Figure H. Fit of MLE estimations for WIKI. The model fit is shown in red and the respective percentile envelope overlay the data, as in Fig 1.

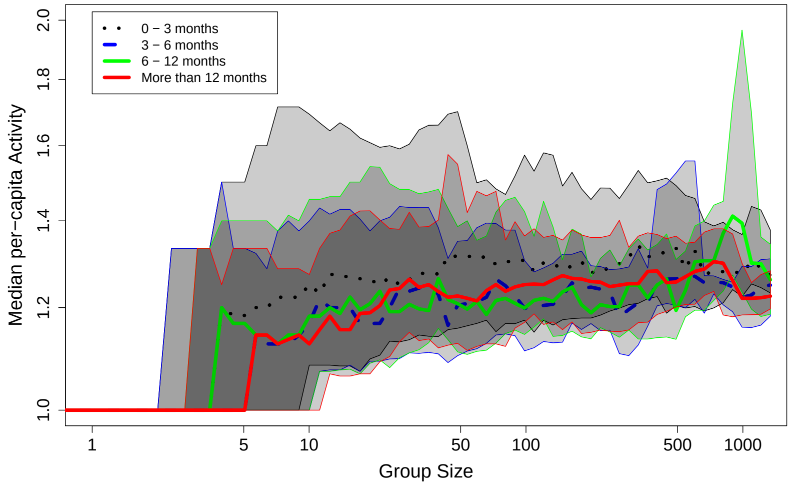

Figure I. Median group mean activity across a range of community sizes in the YOUT data. The median activity is the solid thick line, the shaded areas mark the regions between the 25th to the 75th percentiles.

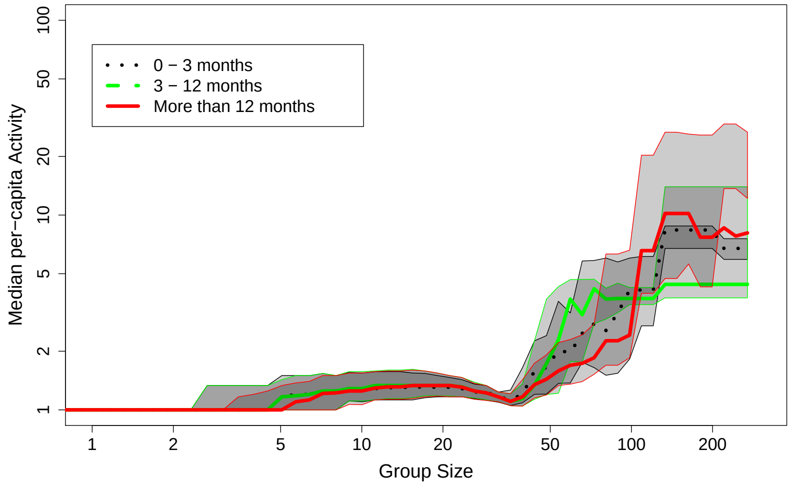

Figure J. Median group mean activity across a range of community sizes in the BRDS dataset. The median activity is the solid thick line, the shaded areas mark the regions between the 25th to the 75th percentiles.
